# The adolescent athlete's heart; A miniature adult or grown‐up child?

**DOI:** 10.1002/clc.23417

**Published:** 2020-07-09

**Authors:** Guido E. Pieles, A Graham Stuart

**Affiliations:** ^1^ National Institute for Health Research (NIHR) Cardiovascular Biomedical Research Centre, Congenital Heart Unit Bristol Heart Institute Bristol UK; ^2^ Institute of Sport, Exercise and Health University College London London UK

**Keywords:** adolescence, athletic adaptation, pediatric arrhythmia, pediatric athlete, pediatric cardiomyopathy, pediatric exercise, sports cardiology

## Abstract

The systematic development of early age talent in sports academies has led to the professionalization of pediatric sport and the sports physician need to be aware of pediatric cardiological problems. Research into the medical cardiac care and assessment of the pediatric athlete are accumulating, but specific pediatric international guidelines are not available yet and reference data for ECG and echocardiography are incomplete, in particular for the age group <12 years of age. This article is an introduction to the physiological and diagnostics specifics of the pediatric athlete. The focus lies in the differences in presentation and diagnosis between pediatric and adult athletes for the most common pathologies. Reference data for electrical and structural adaptations to intensive exercise are sparse particularly in athletes aged below 12 years old. Training related changes include decrease of resting heart rate, increase of cardiac output, ventricular cavity size, and wall thickness. Cardiac hypertrophy is less pronounced in pediatric athletes, as HR mediated cardiac output increase to endurance exercise is the dominant mechanism in peripubertal children. As in adults, the most pronounced cardiovascular adaptations appear in classical endurance sports like rowing, triathlon, and swimming, but the specifics of pediatric ECG and echocardiographic changes need to be considered.

## 1 INTRODUCTION

### 1.1 General activity recommendations for children

Healthy children are more active than adults and exercise and sports are a central experience of childhood and are essential for a normal physical and mental development in health and also chronic disease. Although overall cardiorespiratory fitness has been falling in teenagers for many years, this decline has stabilized since 2000 with the greatest improvement in countries with the least income inequality.^1^, ^2^ The importance of integration of sports and exercise into the daily lives of children is reflected in the international activity recommendations for children and adolescents, modified from References 3, 4.

At least 60 minutes of moderate to vigorous physical activity daily, >60 minutes provide additional health benefits. Most physical activity should be aerobic.

- Vigorous‐intensity activities at least 3 days a week
- Activities that strengthen muscle and bone

These recommendations reflect the overwhelming evidence of health benefits from regular physical exercise and are intended to counteract the alarming sedentary lifestyle in children worldwide. Data from the United Kingdom, suggests, that only 14% of 13 to 15 year old boys and 9% of girls achieve physical activity targets—a figure which has fallen from 28% in boys and 14% in girls in 2008.^5^

In contrast to the sedentary majority, there is an emerging subpopulation of children and adolescents that train to a very high level. Moreover, there is robust evidence to suggest that in children and adolescents, higher amounts of physical activity are associated with multiple health benefits including cardiorespiratory and muscular fitness, bone health, and weight status or adiposity.^6^ Although the term “pediatric athlete” is usually defined as 12 to 16 years old,^7^ regular training can begin from the age of 6 years onward for some sports disciplines (eg, gymnastics). Specific strategies for athlete development from a young age may include “professional” level training intensity and volumes.^8^ However, in addition to some positive adaptive responses, intensive training can have a detrimental effect on the developing musculoskeletal system leading to overuse injuries, overtraining syndrome and burnout.^9^, ^10^ Hence, training the elite pediatric athlete will need to be accompanied by evidenced based medical care for the pediatric athlete.

### 1.2 Epidemiology of disease and sudden cardiac death

The etiology of sudden cardiac death (SCD) in pediatric athletes is comparable to those in young adult athletes. Available data show for example that 2000 children die each year from SCD in the US, the incidence ranging from 0.6‐8/100000.^11^, ^12^ A recent study of 11 168 adolescent footballers, who were undergoing cardiac screening, identified disorders associated with SCD in 0.38% and an incidence of SCD in 6.8 per 100 000 athletes during follow up.^13^ While available data supports the concept that cardiac screening can pick up cardiac disease in pediatric and adolescent athletes,^14^, ^15^, ^16^ the optimal screening strategy is still debated.^17^, ^18^ Additionally its efficacy in preventing SCD in this population is still a matter of debate. The causes of SCD in the pediatric athlete population^19^, ^20^ include 44% structural heart disease such as congenital cardiac anomalies or cardiomyopathies, nonstructural heart disease, primarily channelopathies, accounting for the remaining 56%. The variable etiology, incidence and age of presentation of common causes of SCD such as cardiomyopathies and inherited arrhythmia disorders needs to be taken into account when assessing the true incidence and etiology of SCD in childhood.^21^, ^22^ Alarmingly, sudden cardiac arrest is the first presenting symptom in approximately 50% of cases, providing a strong argument for early detection of disease by preparticipation screening.^23^, ^24^

### 1.3 Maturation, growth, and adaption of the cardiovascular system in childhood

Knowledge of the physiological changes in the pediatric athlete caused by growth, maturation, and training are incomplete but have been increasingly investigated over the last decade. This has informed training and talent development^8^ However, it is still difficult to predict adult athletic performance before puberty.^25^ Many of the most talented young athletes are simply those with those most advanced pubertal development. Trainability and training effects have been investigated in pediatric athletes,^26^ but physiological gender related maturation and development particular during puberty with growth, hormone dependent bone and muscle mass maturation, changes in endurance, speed, strength, balance, age and weight related heart rate, blood pressure and cardiac output, respiratory capacity and peak oxygen uptake need to be taken into account when differentiating training progress from growth and maturation or indeed pathology. Although some sports specialization is important to develop elite‐level skills, it is recommended that intense training in a single sport should be delayed until late adolescence to minimize injury, psychological stress, and burnout.^27^ Exercise training effects in childhood include improvements in bone strength, kinetic skills, biological maturity, lung and endocrine function, academic performance and mental health.^28^

The complex interaction between growth, training, gender and maturity is also true and must be taken into account when assessing the cardiovascular system of pediatric athletes. During puberty, significant changes occur which affect both cardiac size and function and are reflected in screening tests such as echocardiography and the ECG. Importantly, this is also the time where inherited arrhythmia disorders or cardiomyopathies can present for the first time as growth and hormonal changes unmask genetic cardiac disease. This is seen in electrical diseases such as channelopathies and in structural diseases such as the cardiomyopathies. Thus, 23% of children with a family history of Brugada syndrome and a negative ajmaline challenge in early childhood will have a positive (diagnostic) ajmaline challenge if repeated after puberty.^29^ Similarly, in a large multicenter review of young people with catecholaminergic polymorphic ventricular tachycardia (CPVT), the average onset of symptoms was 10.8 years with diagnosis delayed for up to 2.5 years.^30^ This is also relevant for cardiomyopathies (eg, hypertrophic cardiomyopathy [HCM]), where a clear prepubertal phenotype is more difficult to recognize than in adult life.^31^ For children with a family history of HCM, 1 to 2 yearly screening is recommended from aged 10 years^32^ as presentation can occur in childhood.^33^ These factors make the assessment of normality of cardiac size and function challenging, and during childhood, cardiac chamber sizes should be referenced to somatic size using *z* scores or relating cardiac dimensions to height or body surface area. Pubertal stage should also be taken into account, although this is rarely formalized due to the impracticality of assessing pubertal stage in teenagers which requires either an assessment of secondary sexual characteristics or x‐ray imaging such as bone age. Size/gender specific pediatric centiles for both echocardiographic measurements and cardiac MRI in the nonathletic pediatric population are available.^34^, ^35^, ^36^

Prior and La Gerche defined athlete's heart as “the complex of structural and functional electrical remodeling that accompanies athletic training”.^37^ Knowledge and data on physiological changes to athletic training in childhood are well documented in adults but less is known in children and in particular the developing pediatric athlete.^1^, ^38^, ^39^ Studies reporting training‐related cardiovascular changes in peripubertal athletes are summarized in Table 1. Adaptation in cardiac chamber size and myocardial mass have been described,^41^ but are less pronounced in pediatric athletes, which may reflect the different effect of exercise on the immature heart but it may also be due to the reduced intensity and volume of exercise training participated in by younger children. Interestingly, overtraining can occur in pediatric athletes, for example described by elevation in microvolt T wave alternans as a specific sign of overtraining in elite child athletes.^42^ When cardiac adaptations to training occur in the pediatric athlete, the phenotype is different to adults,^43^ These are summarized in Table 2 and include a more pronounced chamber dilatation and less ventricular hypertrophy than in adults.^44^, ^45^

**TABLE 1:** Cardiovascular remodeling in peripubertal child athletes

Study	Number of athletes/sport	Age range	Evaluation technique	Pubertal status described	Conclusions and effect of exercise	Reference
Rowland	14 competitive swimmers; matched active nontrained controls	8.8‐13.5 y (mean 11)	ECG/Echocardiogram	Yes	Lower resting heart rates and LV volume overload in athletes	*Pediatrics* 1987;79(5):800‐804
Telford	85 trained child athletes (mixed) compared with skeletal age matched controls	11‐12 y	Echocardiogram	No	No difference in ventricular dimensions or mass	*J Sports Sci* 1988;6:49‐57
Rowland	10 male runners, matched with active, nontrained controls	11‐13 y	ECG/Echocardiogram/Metabolic exercise testing	yes–described as prepubertal	No clinically significant differences in ECG or LV mass and wall thickness	*Int J SportsMed* 1994;15;515‐519
Ozer	82 swimmers with mean 32 months swim training; 41 sedentary control group	7‐14 y (mean 11.2)	Echocardiography	No	Athletes had increased LV dimensions, wall thickness, aortic root size and LV mass compared to controls	*Jpn Heart J* 1994
Rowland	7 competitive cyclists compared with control group.	11.9 y	Metabolic exercise testing Echocardiography	No	Maximal stroke volume determines VO_2_ max. Lower resting heart rate and higher stroke volume than controls.	*Med Sci Sports Exerc* 2000;32(4):747‐52
Obert	29 boys and girls. 3 months aerobic training/detraining for 2 months (nonexercised control group 26)	10‐11 y	Echocardiography	No	LV internal dimensions increased 4.6% and wall thickness decreased (10.7%) returned to normal after detraining. Heart rate slowed with training. No change in systolic function with training or detraining.	*Int J Sports Med* 2001;22(2):90‐96
Triposkiadis	25 elite swimmers 12‐14 h training per week compared with sedentary controls	11.5 y	Heart rate variability (HRV) Echocardiography	No	Increased vagal dominance, LV and LA dimensions increased. No change in wall thickness or HRV	*Eur J Clin Invest* 2002;32:16‐23
Nottin	12 boy cyclists, 11 untrained controls; 10 adult cyclists and 13 sedentary adults	11–13 y (adults 20‐26 y)	Echocardiography	Yes; Tanner stages. Post pubertal boys excluded.	Increased LV relaxation in adult and child cyclists but no LV hypertrophy in children	*Med Sci Sports Med* 2004;36(9);1507‐1513
Ayabakan	22 male pubertal swimmers compared with 21 age‐matched, sedentary controls. Mean 10 h training per week.	11 y	Echocardiography Including tissue Doppler imaging	Yes (described as prepubertal)	No differences in tissue Doppler but increased concentric LV wall thickness in athletes compared to controls. No change in diastolic dimensions.	*Cardiol in Young* 2006;16:61‐66.
Rowland	7 girls, 7 boys trained swimmers (5 h/week Prone swim simulation. Compared to nontrained controls	12 y (=/− 0.5 y)	Metabolic exercise testing Exercise Echocardiography	No	No rise in stroke volume during exercise implying peripheral factors (increased filling) and heart rate are main determinants of cardiac output on exercise. Minor increase in LV diastolic dimension and mass in trained group.	*J Sci Med Sport* 2009;12:266‐272
Zdravkovic	94 highly trained male footballers	12.85 +/− 0.84 y	Echocardiography	No	Significant increase in LV dimensions, aortic root and LA size	*J Sci Sports Med* 2010;13:602‐606
Koch	342 elite athletes at Sports Schools. Multiple disciplines	10‐15 y‐old	ECG/ echocardiogram	No	LV upper limits described Age 11: boys 10 mm, girls 9 Age 13: boys/girls 10 mm Age 15: boys 11 mm/girls 10 mm. No ECG gender differences	*Eur J Prev Cardiol* 2014;21(6):774‐781
Binnetoglu	140 athletes; 6 Sports minimum 3 h per week for 2 y, sedentary controls	10‐16 y	ECG/echocardiogram including strain imaging	No	Normal systolic and diastolic indices in athletes. 16% concentric remodeling; 28% eccentric remodeling. Strain lower in athletes. Myocardial deformation more evident in mixed sports participants.	*Pediatr Cardiol* 2014;35:126‐139
Agrebi	Elite male national handball players; male. 3 groups of 12	Mean age 12/16/25 y	ECG/echocardiogram	No	Chamber dilatation occurred in younger athletes but less hypertrophy compared to older athletes.	*PLoS ONE* 2015;10(12):e0143609. doi:10.1371/journal.pone.0143609
Calo	2261Caucasian male soccer players	Mean age 12.4 y	ECG/Echocardiogram	No	Anterior T wave inversion (>2 leads) associated with cardiac disease in 4.8%: T wave inversion (inferolateral leads) associated with disease in 60%	*Heart* 2015;101;193–200

*Note:* Reproduced with permission from Reference 40.

**TABLE 2:** Cardiovascular adaption to exercise training in child athletes‐comparison with adults

Cardiovascular change in child	Comparison with adult athletes	Comment
Resting heart rate falls	Resting heart rate remains higher than in adult	Age‐dependent. Younger athletes have higher resting heart rates
Dilatation of left atrium	Similar pattern	Considerable variation between children in the same exercise group and in different studies. Some studies have demonstrated concentric hypertrophy, others predominantly dilatation. If LV dilates above 60 mm in diastole consider pathology.
Left ventricle dilates, mild LV hypertrophy	Less chamber dilatation and more hypertrophy occurs in adults	
Mild concentric hypertrophy with prolonged vigorous training	Eccentric hypertrophy tends to occur in adults with athletes heart	
Increased LV relaxation Improved diastolic function	Similar pattern	Occurs in prepubertal and post pubertal children
Raised VO_2_ max in comparison to untrained	Lower VO_2_ max relative to body size in comparison to adult athletes	Reflects lower maximal stroke volume and maturity related increase in diastolic filling
Reduced vascular stiffness	Similar pattern	Acute effect known but long‐term effects not studied in children
No differences between the sexes	Female athletes have higher resting heart rates, smaller cardiac chambers, and less hypertrophy	Prepubertal changes present but change to adult pattern post puberty

*Note:* Reproduced with permission from Reference 40.

## 2 DIAGNOSTIC ASSESSMENT OF THE PEDIATRIC ATHLETE

Current data are insufficient to provide evidence‐based guidance on when and how often to screen the pediatric athlete. In Italy, it has been the practice for 40 years to screen all child athletes over 13 years old every 2 years using an ECG and since 2005, the European Society of Cardiology have recommended that two yearly screening should take place using a preparticipation questionnaire, physical examination, and ECG from the start of competitive activity—“usually aged 12‐14 years”.^46^ However, although the overall outcome of such programs has been to significantly reduce the incidence of athletic related death there are few data on the efficacy of this screening process in younger athletes. It is established, that while SCD is a rare event, inherited diseases can present and be diagnosed or indeed excluded by experienced pediatric cardiologists. Although the current recommendations are based on overwhelmingly adult data,^47^ general guidance is provided by the Association for European Pediatric and Congenital Cardiology (AEPC) working group on Sports Cardiology, Physical Activity, and Prevention document,^48^ additionally it is consensus that the current international recommendations for adult athlete ECG screening should be followed.^49^ Despite age and gender specific data on cardiac adaptation it remains particularly important to have an individualized approach not only to training,^8^ but also to cardiac assessment of the pediatric athlete as pace of growth and maturation of the cardiovascular system show significant interindividual differences.

### 2.1 Medical, Family history, and physical examination

Medical and family history should include an accurate documentation of sports and exercise training history. It is important to include any school or college ‐based sports activities which often co‐exist with the child athlete's main sport. An assessment of training volume and intensity and sporting discipline should be made. Family history is very important to help detect congenital, inherited or acquired cardiac disease and risk factors (dyslipidemia, hypertension, and diabetes). Medical history will detect symptoms such as palpitations, exercise dependent respiratory symptoms, dizziness, and syncope and recent infections. Based on physician experience, pyrexia, should result in a training break, whereas in simple upper airway only infection light endurance training can be continued^50^, ^51^ Nutritional assessment should be included, also as anorexia is relatively common especially in sports with a female predominance,^52^ and nutritional supplements such as energy drinks can be arrhythmogenic by adrenergic overstimulation.^53^ Examination will investigate for congenital lesions (specific heart murmurs or pulse, BP differences in hand and feet (eg, coarctation). Additionally, particularly relevant in paralympic athletes is the assessment for syndromes, many are associated with cardiac disease (eg, signs of connective tissue disease, Marfan syndrome, Williams syndrome, and Turner syndrome).

### 2.2 12‐lead ECG

**FIGURE 1:**
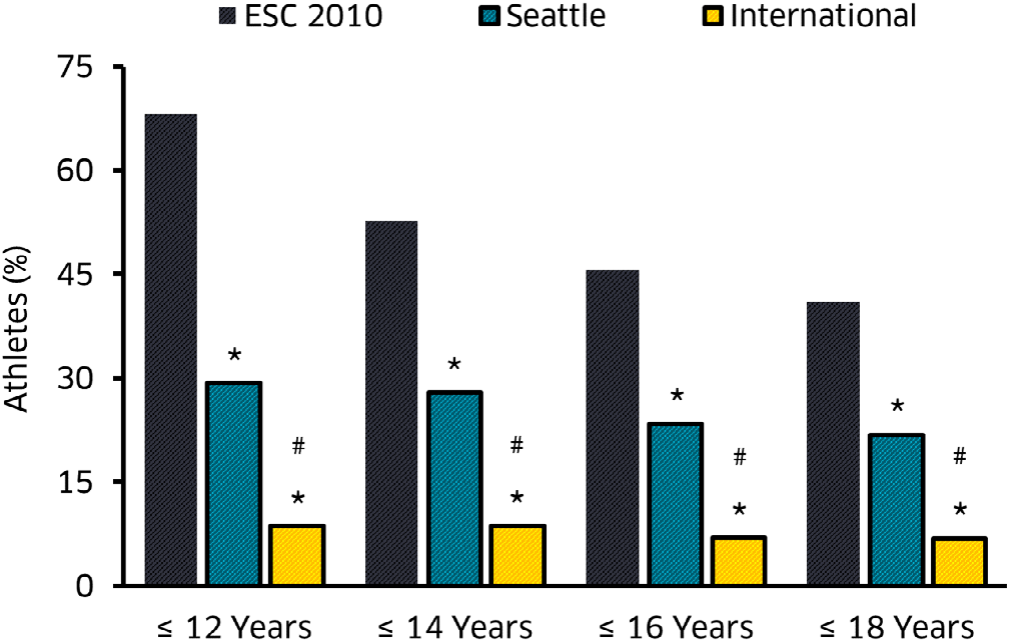
Bar chart shows the percentage of false‐positive ECG findings according to the 3 ECG interpretation criteria by chronological age. *P < .05, significantly reduced prevalence to ESC 2010 recommendations. #*P* < .05, significantly reduced prevalence to Seattle Criteria, reproduced with permission from Reference 61

The 12‐lead ECG is the primary cardiac screening tool in athletes.^49^ Moreover, even in the USA where the ECG is less commonly used as an athletic screening tool, many pediatricians will use the ECG selectively for assessment rather than relying on clinical examination and preparticipation questionnaire alone,^54^ therefore detailed knowledge of the specifics of the pediatric ECG is required when assessing pediatric athletes. For healthy children, interpretation follows routine age and height dependent reference values^55^ with respect to heart rate, time intervals, heart axis, negative precordial T waves, and signs of hypertrophy. The ECG during early childhood gradually moves from the right dominant infancy pattern to the typical adult appearance at the end of puberty. Normal centiles for the childhood ECG are available.^56^ Prepubertal T wave inversion (TWI) in right precordial leads is common and while anterior TWI (V1‐V3) in an adult is often abnormal and may suggest pathology such as arrhythmogenic right ventricular cardiomyopathy, identical T wave inversion can be normal in a young teenager and was found in 8.4% of under 14 years old but only in 1.7% over 14 years olds with the only predictor being incomplete pubertal status.^57^ Other studies in caucasian soccer players (mean 12 years, range 8‐18) found TWI present in 136 (6%), virtually always in anterior leads (>90%). Anterior TWI was associated with mild cardiac disease in 4.8% but infero‐lateral TWI was associated with LH hypertrophy or cardiomyopathy in 60%.^58^ TWI in V1 to V3 is common in adolescent caucasian athletes (<16 years) but only 0.1% had TWI beyond V2 after 16 years .^59^ Recent meta‐analysis data show that ECG adaptation such as voltage criteria for atrial and ventricular hypertrophy and repolarization changes occur in athletes as young as 12 years and TWI in chest leads beyond V3 (extended anterior leads to V4) is only found in a minority of healthy 14 to 16 year old athletes.^60^ No specific recommendations for the ECG interpretation in pediatric athletes exist, and the current ECG interpretation guidelines for adult athletes^49^ should be followed. McClean demonstrated these recommendations to have an acceptable overall accuracy in pediatric athletes compared to previous adult ECG recommendations^61^ (Figure 1 **)**. This was confirmed by Malhotra although in this study older teenagers were investigated with mean age of 16.4 years.^62^ The international recommendations, however, are valid only from 14 years of age and while chronological age of the athlete helps to differentiate between a physiological juvenile and pathological T wave morphology, biological age is a more reliable denominator.^63^

### 2.3 Echocardiography

Echocardiography is the first line diagnostic imaging tool also for the pediatric athlete. Pediatric echocardiographic examinations should include a morphological assessment^64^ and include a coronary artery assessment.^65^ Normative reference values exist for cardiac morphology^66^ and function^67^ in the nonathlete pediatric population, values should be indexed for BSA and z‐scored. Reference data for the healthy paediatric population for advanced echocardiographic parameters are available for 2‐D strain ^68^ or exercise stress echocardiography.^69^, ^70^ Recently, studies have investigated pediatric athlete specific morphologic echocardiographic parameters for the LV,^71^ RV,^72^, ^73^ but also atria^74^ and complement previous studies in adolescents athletes providing evidence of quantitative wall thickness and cavity size athletic adaptation.^45^, ^75^, ^76^ Comprehensive data correlating echocardiographic parameters to sports discipline, training volume and intensity, ethnicity, gender and age are still missing for the pediatric age group.

### 2.4 Cross sectional imaging

Cross sectional imaging to investigate pediatric athletes for a cardiac pathology should follow adult recommendations,^77^ but protocols (higher incidence of undiagnosed congenital heart disease in children) and the relevance of specific diagnostic parameters (different diagnostic criteria for cardiomyopathies) differ. Size and gender specific pediatric centiles are available for cardiac magnetic resonance imaging (CMR) parameters.^34^, ^35^, ^36^ CMR has recently been used as a primary screening tool for cardiac anomalies, in particular coronary artery anomalies.^78^ Cardiac CT is the imaging modality of choice in the delineation of small anatomical structures such as coronary arteries and collateral arteries and for imaging parenchymal lung pathology.

### 2.5 Exercise assessment

Exercise testing, in particular cardio‐pulmonary exercise testing (CPET) is also a core diagnostic tool in children to investigate metabolic, respiratory, or cardiac pathology^79^, ^80^ as it can investigate pulmonary vascular disease, dysfunction of the autonomic nervous system, peripheral defects in oxygen transport, oxygen utilization at the working muscle and indirectly cardiac function. It can be safely performed in children above the age of 8 years.^81^ CPET should always be combined with exercise 12‐lead ECG testing to allow assessment of underlying exercise‐related arrhythmias. It is paramount that supervising personnel should be trained and familiar with the pediatric population to encourage maximal effort and deliver reliable test results.^82^, ^83^ Exercise metabolism in pediatric athletes differs from adults in that children do not always attain a true VO_2_ max,^84^ and submaximal measures such as ventilatory anaerobic threshold^85^ or oxygen uptake efficiency slope^86^ can be equally good markers for endurance performance. CPET is also ‐ used to indirectly investigate stroke volume response in pediatric patients with ventricular dysfunction, cardiomyopathies or congenital heart disease and is a core part in assessing sports participation eligibility also in the pediatric athlete. Exercise stress echocardiography can aid in detecting early systolic and diastolic dysfunction, assess valve function, myocardial exercise reserve, subtle wall motion abnormalities or dyssynchrony. Myocardial exercise reserve to investigate borderline or low cardiac function at rest, often seen in athletes can be determined by relatively load independent parameters such myocardial deformation (strain) and Tissue Doppler where, contrary to the adult population, normative reference data exist.^69^, ^70^ No studies exist to date using exercise stress CMR to assess cardiac volumes, function and wall motion abnormalities during exercise in athletes as is now performed in adult athletes.^87^ Cableless mobile and app based devices to monitor heart rate and rhythm during exercise have been piloted in children and are a credible alternative to laboratory testing.^88^

## 3 SPECIFIC DIAGNOSTIC CONSIDERATIONS IN THE PEDIATRIC ATHLETE

The spectrum of hereditary and acquired arrhythmia disorders and cardiomyopathies as a cause of SCD is very similar to that seen in adult athletes, and thus, adult competitive sports participation guidelines should be adhered to in the diagnosis and management of arrhythmias^89^ and cardiomyopathies.^90^ In pediatric athletes however, age and mode of presentation can be different from that in adult for arrhythmias and cardiomyopathies. Genetic testing in index cases and family cascade genotyping in pediatric cardiomyopathies should be performed when clinically indicated. However, age of independent‐ and legal‐capacity to consent to genetic testing is debated and a close collaboration with a geneticist and genetic counselor is recommended. No specific pediatric guidelines for pediatric cardiomyopathy genetic testing exist, but there is emerging evidence, that genotyping can help in risk stratification (reviewed in Reference 91). In all pediatric athletes with a suspected cardiomyopathy, serial annual evaluation is paramount and should include ECG, echocardiogram, exercise testing, and CMR.

#### 3.1.1. Hypertrophic cardiomyopathy

Family and medical history are central as the majority of cases remain phenotypically dormant in childhood and first line 12‐lead ECG screening should follow the published international ECG criteria for athletes. While anterior T wave inversion is related to mild cardiac disease in 4.8%, infero‐lateral T wave inversion in 60% with cardiomyopathy or LVH.^58^ Family history, ECG, Echocardiography, and CMR are imaging tools of choice. LV hypertrophy should be categorized as abnormal if the LV end‐diastolic wall thickness *z*‐score >2. End‐diastolic LV diameter is reduced in the majority of phenotypic pediatric HCM,^92^ however, more common are mild and developing or subclinical phenotypes in childhood. As in adults, abnormal echocardiographic diastolic tissue Doppler parameters or myocardial systolic strain can be first indicators of an evolving phenotype.^92^ Data on the use of de‐training in the pediatric athlete population to ascertain a diagnosis of HCM is still absent. Risk factors for cardiac events in the nonathlete pediatric HCM population are those of adults, but a pediatric risk stratification model has been recently suggested.^93^

#### 3.1.2. Arrhythmogenic ventricular cardiomyopathy

Disease is often concealed in childhood and mimics changes seen in the healthy young athletic population.^94^ Diagnostic criteria are less sensitive and specific in pediatric ARVC^95^, ^96^ as they are based on data from affected adults^97^ Echocardiographic revised task force criteria in particular rarely trigger suspicion in children and adolescents as they rely on adult RV diameters and are less sensitive and specific in pediatric ARVC^98^ and enlarged RV diameters in pediatric athletes can mimic cardiomyopathic changes.^73^ Recent data suggest that additional modalities such as 2‐D strain are more sensitive in assessing adolescents with ARVC.^96^ CMR is the diagnostic imaging modality of choice also in children, but CMR findings in adult arrhythmogenic ventricular cardiomyopathy (AVC) such as fatty infiltration and fibrosis are of limited value in children and focus lies on ventricular function, regional wall motion abnormalities, and *z*‐scores of RV and LV dimensions.^95^ Arrhythmic activity is highly variable in adolescent patients with AVC,^99^ serial arrhythmia monitoring and exercise testing is important, LV involvement in adolescents is less frequent. Exercise can unmask or exacerbate AVC disease and pediatric athletes with phenotypic AVC should be restricted following adult recommendations.^90^

#### 3.1.3. Left‐ventricular noncompaction cardiomyopathy

Left‐ventricular noncompaction cardiomyopathy (LVNC) in childhood shows an undulating and heterogeneous phenotype of different severity.^100^ LV hypertrabeculation is not uncommon in the healthy adult athlete,^101^ and this is similar in the pediatric population,^78^ however, a definite differentiation between physiological trabeculation and LVNC disease in this age group remains a challenge. Mild hypertrabeculation in the setting of normal function, without CMR features such as wall motion abnormalities or fibrosis, and without evidence of rhythm abnormalities can be regarded as a normal phenomenon, but serial monitoring is advised. Advanced echocardiographic imaging tools such as speckle tracking strain imaging can help detect disease in childhood,^102^ but cross section al imaging and arrhythmia monitoring is warranted.

#### 3.1.4. Pre‐excitation in the pediatric athlete

Ventricular pre‐excitation appearing as a delta wave on the ECG, occurs in children and may be symptomatic (Wolff Parkinson White syndrome) or asymptomatic and may be constant or intermittent. Although 65% of adolescents are asymptomatic, in comparison to 40% of adults, even asymptomatic young people may experience a sudden death event with no prior symptoms.^103^, ^104^ Moreover, although it used to be thought that intermittent pre‐excitation was a good prognostic finding, it is now clear that intermittent preexcitation does not imply a low risk pathway in children.^105^ Consequently, most pediatric electrophysiologists would now carry out an invasive electrophysiology study to establish risk and the presence of pre‐excitation on the ECG of a child athlete should lead to a referral to a pediatric electrophysiologist for further risk stratification.^106^

### 3.2 Congenital heart disease

Congenital heart disease (CHD) is the most common birth defect with a good survival rate and there are now many more adults than children with congenital heart disease—approximately 1 in 150 young adults have some form of CHD.^107^ Recently, exercise advise in children and adolescents has shifted away from a more restrictive approach. It is now recommended that exercise advice and prescription should be an integral component of every patient encounter.^108^ The assessment and follow up of the pediatric athlete should be in the hands of experienced congenital cardiologists, the ‐ recommendations on individual exercise and eligibility assessments in ‐ patients with CHD help risk stratify individuals in terms of type, intensity and level of exercise^109^ but these relate to athletes over the age of 16 years of age.

### 3.3 Care of the pediatric athlete in the future

The rapid development and professionalization of youth sports academies demands increased diligence to safeguard the development of the pediatric athlete. The significant progress achieved in adult sports cardiology can guide assessment of the pediatric athlete, but adult cardiac preparticipation screening and management recommendations cannot be unequivocally applied to the pediatric heart. Somatic growth, psycho‐cognitive maturation with particular communicational and also ethical and legal considerations require an interdisciplinary approach involving pediatricians, pediatric cardiologists and sports medicine professionals as well as coaching staff with the young athlete in the center.

Future research should focus on the development of gender, growth, and age specific normative assessment criteria for ECG and echocardiogram to increase diagnostic accuracy. Most importantly, development of training pathways and a stronger engagement of pediatric and sports governing bodies is required, as too few pediatricians and pediatric cardiologists are sufficiently trained to provide expert opinion on the pediatric athlete.^110^ Consequently, an approach requiring synergy between pediatric and sports cardiologists, exercise physiologists, policy makers, and sports organizations is required to develop pediatric cardiac monitoring tools and protocols, eventually working towards a child athlete centered specialty (pediatric sports cardiology) that matches the sports professionalism of the current and future pediatric athlete.

## CONFLICT OF INTEREST

Dr. Guido E. Pieles is director of the sports cardiology consulting company “Cardiac Health and Performance Ltd”, Prof. A Graham Stuart is Medical Director of “Sports Cardiology UK”—a company which specializes in the assessment and management of athletes with cardiovascular problems.

## ETHICS STATEMENT

This article does not contain any studies with human participants or animals performed by any of the authors.
